# Signal strength controls the rate of polarisation within CTLs during killing

**DOI:** 10.1083/jcb.202104093

**Published:** 2021-07-22

**Authors:** Gordon L Frazer, Christian M Gawden-Bone, Nele M G Dieckmann, Yukako Asano, Gillian M Griffiths

**Affiliations:** 1 Cambridge Institute for Medical Research, Biomedical Campus, Cambridge CB2 0XY UK

## Abstract

Cytotoxic T lymphocytes (CTL) are key effector cells in the immune response against viruses and cancers, killing targets with high precision. Target cell recognition by CTL triggers rapid polarisation of intracellular organelles towards the synapse formed with the target cell, delivering cytolytic granules to the immune synapse. Single amino acid changes within peptides binding MHC class I (pMHC) are sufficient to modulate the degree of killing, but exactly how this impacts the choreography of centrosome polarisation and granule delivery to the target cell remains poorly characterised. Here we use 4D imaging and find that the pathways orchestrating killing within CTL are conserved irrespective of the signal strength. However, the rate of initiation along these pathways varies with signal strength. We find that increased strength of signal leads to an increased proportion of CTL with prolonged dwell times, initial Ca^2+^ fluxes, centrosome docking and granule polarisation. Hence TCR signal strength modulates the rate but not organisation of effector CTL responses.

## Introduction

Cytotoxic T lymphocytes (CTL) are the key effector cells killing targets with high precision. This is achieved through the specificity of the clonally expressed T cell receptor (TCR) for a specific peptide chain loaded into a major histocompatibility complex (pMHC). TCR-pMHC binding triggers signalling and induces the CTL to polarise and dock the centrosome at the point of TCR signalling, allowing precise delivery of cytotoxic granules. Recent studies have demonstrated that TCR signals modulate membrane changes and reorganisation of the actin cytoskeleton at the point of secretion (Gawden-Bone et al., 2018; Ritter et al., 2015; Ritter et al., 2017). However, little is known about how the many steps required for intracellular polarisation of the secretory machinery within CTL change with the strength of TCR signal.

The OTI transgenic mouse provides a particularly well understood system for examining the effects of altering the strength of TCR signalling. All T cells in OTI transgenic mice express a clonal TCR recognising peptides from ovalbumin, with the canonical peptide that binds MHCI (H2K^b^) being SIINFEKL (OVA257-264) (Hogquist et al., 1994; Kelly et al., 1993; Koniaras et al., 1999). Single amino acid changes in this peptide, termed altered peptide ligands (APL), have been used to alter CTL killing within a population of cells. This OTI system is exceptionally well described, with the strength of pMHC-TCR interactions understood at the structural level (Denton et al., 2011) via studies describing the Kon/off rates and strength of TCR-MHC interactions for different APLs (Alam et al., 1996; Alam et al., 1999; Naeher et al., 2007) (Huang et al., 2010) (Liu et al., 2014). The impact of varying strength of interactions upon CTL activation (King et al., 2012; Naeher et al., 2007; Ozga et al., 2016; Palmer et al., 2016; Zehn et al., 2009; Zehn et al., 2014) and killing efficiency is also well characterised within CTL populations (Daniels et al., 2006; Hogquist et al., 1995; Hogquist et al., 1994; King et al., 2012; Ozga et al., 2016; Zehn et al., 2009). Previous studies using this system have shown a reduction in the number of conjugates formed between CTL and targets (Jenkins et al., 2009; Palmer et al., 2016; Yachi et al., 2006), and suggested that granule polarisation was reduced with lower affinity ligands(Jenkins et al., 2009). However, these studies examined a snap-shot of events within a population of cells at fixed time-points. Little is known about how varying strength of TCR signalling controls the polarisation and delivery of killing within individual CTL within the population.

Several possible mechanisms exist by which stimulation strength might affect killing. First, the co-ordination of each of the different stages required for successful killing (attachment, centrosome and granule polarisation) could change with signal strength; for example, centrosome polarisation to the synapse might occur without concomitant granule polarisation (Beal et al., 2009; Jenkins et al., 2009). Conversely, the speed of individual stages within the process might change; for example, the speed of centrosome movement might be dictated by strength of signal. Alternatively, the co-ordination of individual steps might be modulated in rate but not organization by stimulation strength, as was previously observed during activation of naïve T cells (Ma et al., 2020; Richard et al., 2018). Here we use high-resolution 3D and 4D spinning disk confocal microscopy and image analysis to interrogate the stages of CTL killing and hence distinguish these possibilities, revealing how CTL killing efficiency is controlled.

## Results

### Increasing TCR signal strength increases the time CTL dwell on a target

We used a lactate dehydrogenase (LDH) release assay to measure target cell death. Consistent with previous studies, we found that increasing TCR signal strength by varying APL to increase TCR-pMHC affinity resulted in increased killing (Daniels et al., 2006) (Supplementary Figure 1a). This increase in killing efficiency mirrored the increase in CTL degranulation (Supplementary Figure 1b-d).

**Figure 1:**
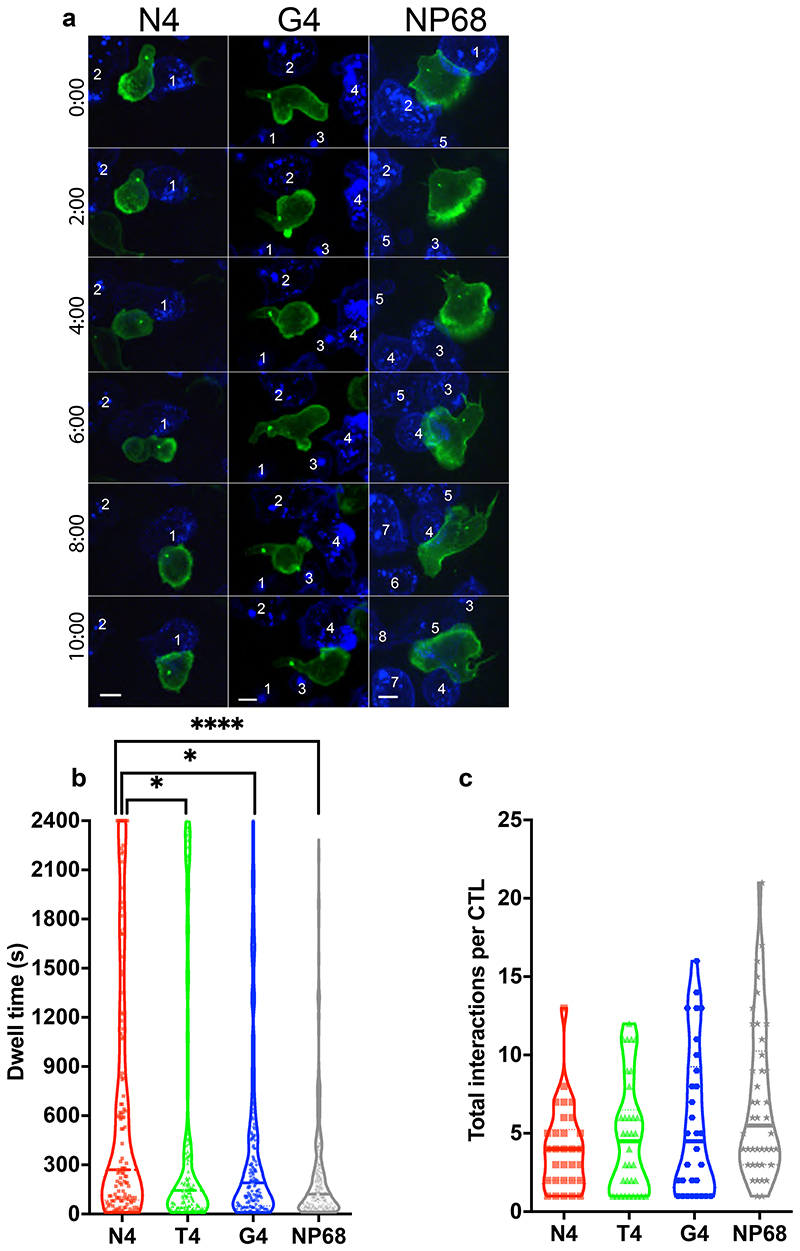
Increasing TCR signal strength increases CTL dwell time. OTI CTL expressing Lifeact-mApple (green) and RFP-PACT (green sphere) interacting with EL4 (blue), pulsed with N4, T4, G4 or NP68 peptides (a) Representative timeseries of OTI CTLs encountering targets, numbered sequentially. Scale bars = 5μm (b) Violin plot showing dwell times for individual CTLs with targets; number of interactions N4 n=121, T4 n=128, G4 n=169, NP68 n=294; bars represent median with quartiles. A Bonferroni corrected Mann-Whitney test was used for statistical analysis with *=p<0.5 and ****=p<0.0001. (c) Data from (b) was used to calculate the mean number of interactions per CTL per time-series, and plot the mean per independent movie (N4 n=30, T4 n=34, G4 n=30, NP68 n=42). Bars represent median with quartiles.

In order to establish how signals of increasing strength increased killing, we first investigated how signal strength affected the length and number of contacts between CTLs and target cells. Using spinning-disk microscopy to image live CTLs interacting with targets in 3D (4D imaging) we found a significant increase in dwell time with higher affinity ligands (Figure 1a,b, Video 1). Furthermore, as TCR signal strength was increased, the mean number of CTL target interactions per CTL decreased, with a mean of 5.63 (+/- 4.75 SD) interactions per CTL for targets pulsed with the weakest APL (G4) over 40 minutes to 4.77 (+/- 3.61 SD) for T4 (mid), and only 4.03 (+/- 2.66 SD) for N4 (strong) (Figure 1c). These data indicate that CTL-target cell interactions stimulated by higher affinity ligands give rise to a more homogeneous response with CTL contacting fewer targets for longer times.

### Loss of charge and the resulting depletion of actin from the synapse is impaired with weak TCR signalling

**Figure 2:**
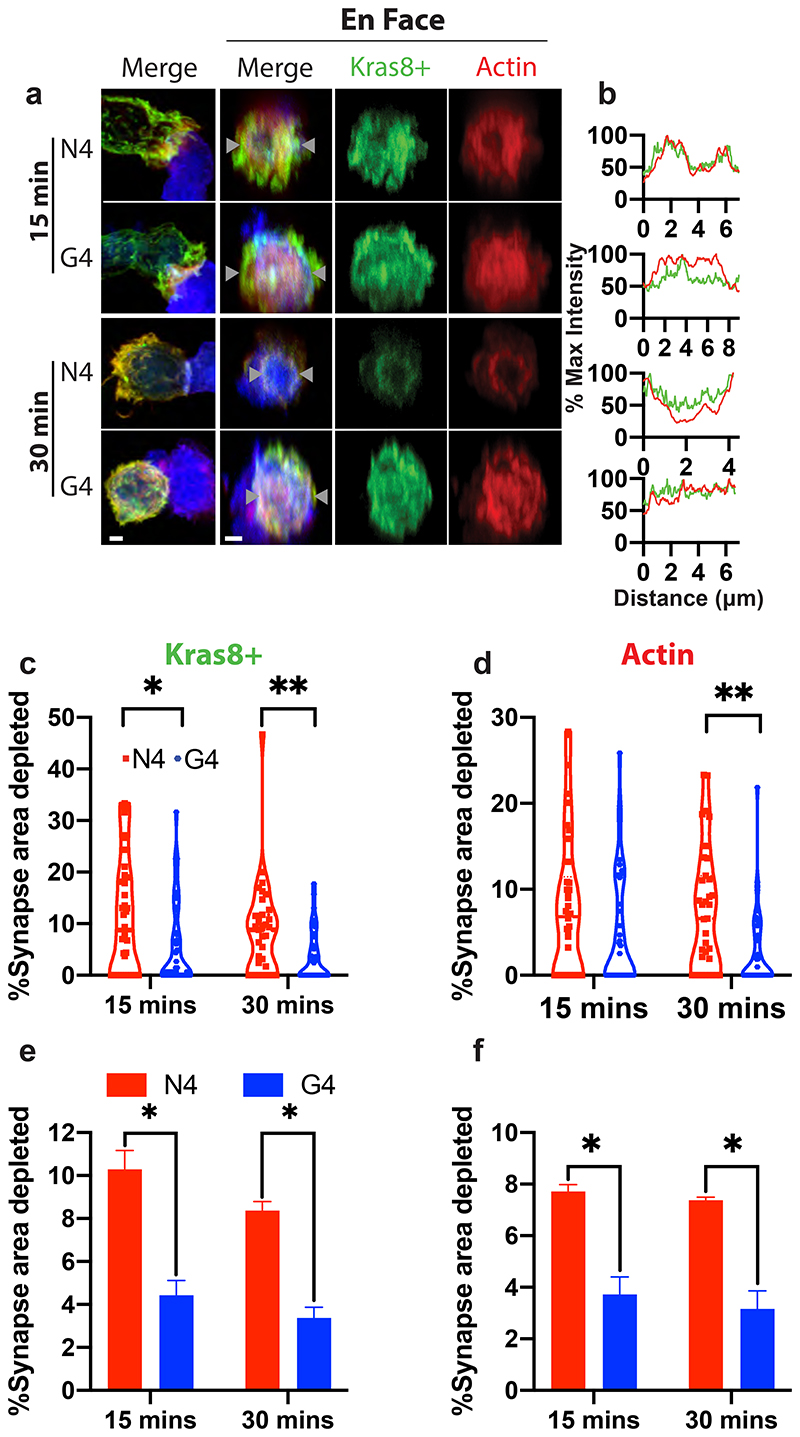
Increasing TCR signal strength increases actin depletion. Confocal projections showing cell conjugates or 3μm depth *en face* reconstructions across the synapse for OTI CTL expressing Kras8+ probe (green) conjugated with N4 or G4 presenting EL4 for times as shown prior to fixation and labelling for actin (red). (a) Representative images of conjugates showing actin (red), Kras8+ in (green), and EL4 (blue). Maximum intensity projections (left), and *en face* projections (right) for merged, actin and Kras8+ signals. Scale bars = 1μm. (b) Intensity profiles for actin and Kras8+ along line marked between grey triangles. (c,e) Area of synapse depleted for Kras8+ expressed as a percentage of the total synapse area taken from the *en face* images for (c) each individual cell in one representative experiment (n=30 per condition), (e) the mean, bars show SEM from 3 separate repeats. (d,f) Area of synapse depleted for actin as a percentage of the total synapse area taken from the *en face* images for (d) each individual cell in one representative experiment (n=30 per condition), (f) the mean, bars show SEM from 3 separate repeats. A two-tailed Bonferroni corrected Mann-Whitney test was used for statistical analysis of (c-f) with *=p<0.5 and **=p<0.01.

Previous studies have shown that actin controls granule release at the cytolytic synapse, with phosphatidylinositol 4,5-bisphosphate (PIP2) required for actin recruitment across the synapse (Ritter et al., 2017). Upon TCR signalling PIP2 is rapidly cleaved to generate diacylglycerol (DAG). This results in both the loss of actin recruitment and changes the charge across the membrane, preventing phosphatidylinositol-4-phosphate 5-kinase (PIP5K) association required to replace PIP2 (Gawden-Bone et al., 2018). To examine if TCR signal strength modulates the loss of negative charge and depletion of actin from the synapse, we used OTI CTL nucleofected with an EGFP-Kras8+ probe to detect membrane negative charge, along with phalloidin F-actin and anti-phosphotyrosine to identify synapses where signalling had occurred in fixed conjugates. Using en-face image analysis and quantitation we found a greater depletion of both membrane charge and actin across the synapse in conjugates formed with strong (N4) compared to weak (G4) affinity ligands (Figure 2a-d). The mean area of both charge and actin depletion increased with the higher affinity ligand (N4) compared to the weak ligand (G4) (Figure 2e,f). These depletions showed similar results with conjugates formed for 15 or 30 minutes. Thus, stronger TCR signals resulted in an increased frequency of long-term interactions between CTL and targets. In addition, stronger signals promoted an increased proportion of CTL depleting actin across the synapse, in keeping with the molecular mechanism of actin depletion proposed^3^. As increased dwell times are likely to result in sustained signalling it is possible that these two changes work in concert.

### Increasing TCR signal strength enhances centrosome polarisation to the synapse

**Figure 3:**
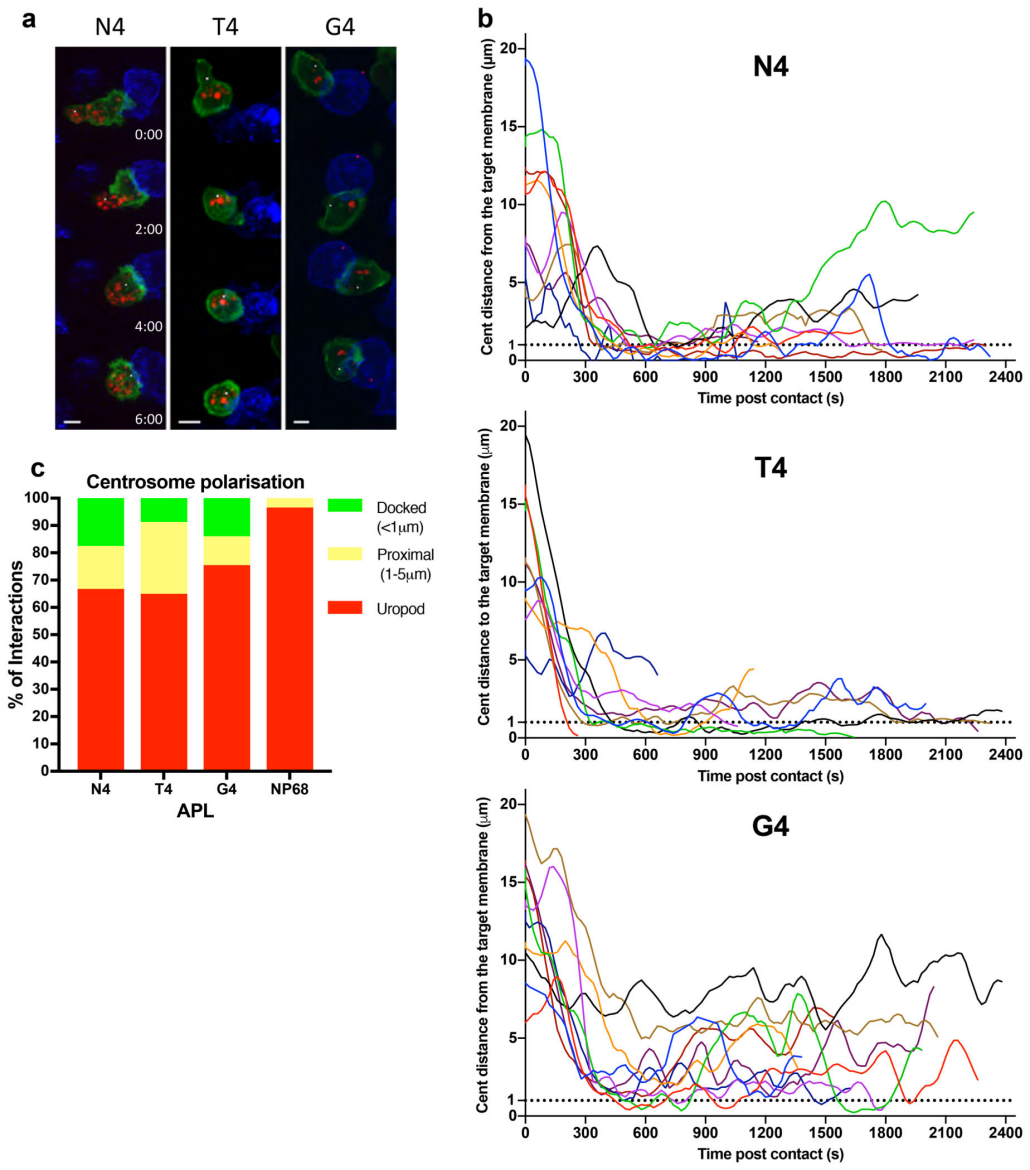
Increasing TCR signal strength increases centrosome docking at the synapse. GzmB-TdTomato (red) OTI CTL expressing Lifeact-EGFP (green) and BFP- PACT (white sphere) interacting with EL4 (blue), pulsed with N4, T4, G4 or NP68 peptides. (a) Representative max-intensity projection timeseries of OTI CTLs encountering targets. Scale bars = 5μm, time min:sec post contact with target. (b) Segmented centrosome distances to synapse measured across the duration of the interaction for n=10 (N4, G4) or n=9 (T4) independent CTL- target interactions, with each colour representing a different CTL. (c) OTI expressing LifeAct-mApple and RFP-PACT were imaged every 10s over 40 minutes interacting with EL4-blue, pulsed with N4, T4, G4 or NP68. The closest approach of the centrosome to the target cell membrane per interaction was classified as in the uropod (>5μm), proximal (1-5μm) or docked (<1μm). Results from 5 independent experiments, with CTL N4=58, T4=57, G4=80, NP68=126 and total interactions analysed: N4=121, T4=129, G4=169, NP68=284.

Polarisation of the centrosome to the synapse plays a crucial role in directing degranulation of cytolytic components toward the target cell. We used the PACT-domain, a marker that localises to the centrosome (Gillingham and Munro, 2000), to follow centrosome polarisation to the synapse as CTL formed conjugates with targets (Figure 3, Video 2). Tracking the centrosome within individual CTLs that formed a stable conjugate with a target cell, we found that with N4 stimulation, the centrosome traversed the cell to dock at the synapse (defined as <1μm from the synapse) within 600s in 80% of conjugates and the centrosome had docked in 100% of conjugates by 750s. In contrast the centrosome docked in only 55% of conjugates by 600s for T4 with only 66% docking by 750s. In response to G4 stimulation centrosome docking was only seen in 20% of conjugates by 600s increasing to 30% by 750s. Of note, at 900s the centrosome was docked in 60% N4, 33% T4 and 0% G4 of conjugates (Figure 3a,b). This suggests that with higher affinity ligands, the frequency of cells with docked centrosomes at the synapse increases with TCR signal strength.

We next asked whether the speed of centrosome movement varied, but found no substantial differences in the timing or maximum speed with which centrosomes docked at the synapses when stimulated by N4, T4 or G4 pMHC interactions (Supplementary Figure 2). Fluctuations were observed post-docking after stimulation with G4, in keeping with the variations in centrosome distance from the synapse observed with G4 that suggest docking was transient and less stable with weaker signals (Figure 3b). Thus, the speed of centrosome movement appeared constant, but the frequency of CTL with docked centrosomes increased with TCR signal strength.

We verified this by measuring the closest centrosome distance to the synapse in 3D live imaging of a population of CTLs interacting with targets over a 40-minute period, including transient interactions (Figure 3c). We found that centrosome docking occurred in some CTLs regardless of stimulation strength, albeit only transiently with weaker signals. Only when an irrelevant peptide (NP68) was used was no docking seen. Thus, while centrosome docking occurred regardless of signal strength, the rate at which centrosome docking was achieved within the CTL population varied according to signal strength.

### Docking of the centrosome promotes simultaneous delivery of granules to the synapse

**Figure 4:**
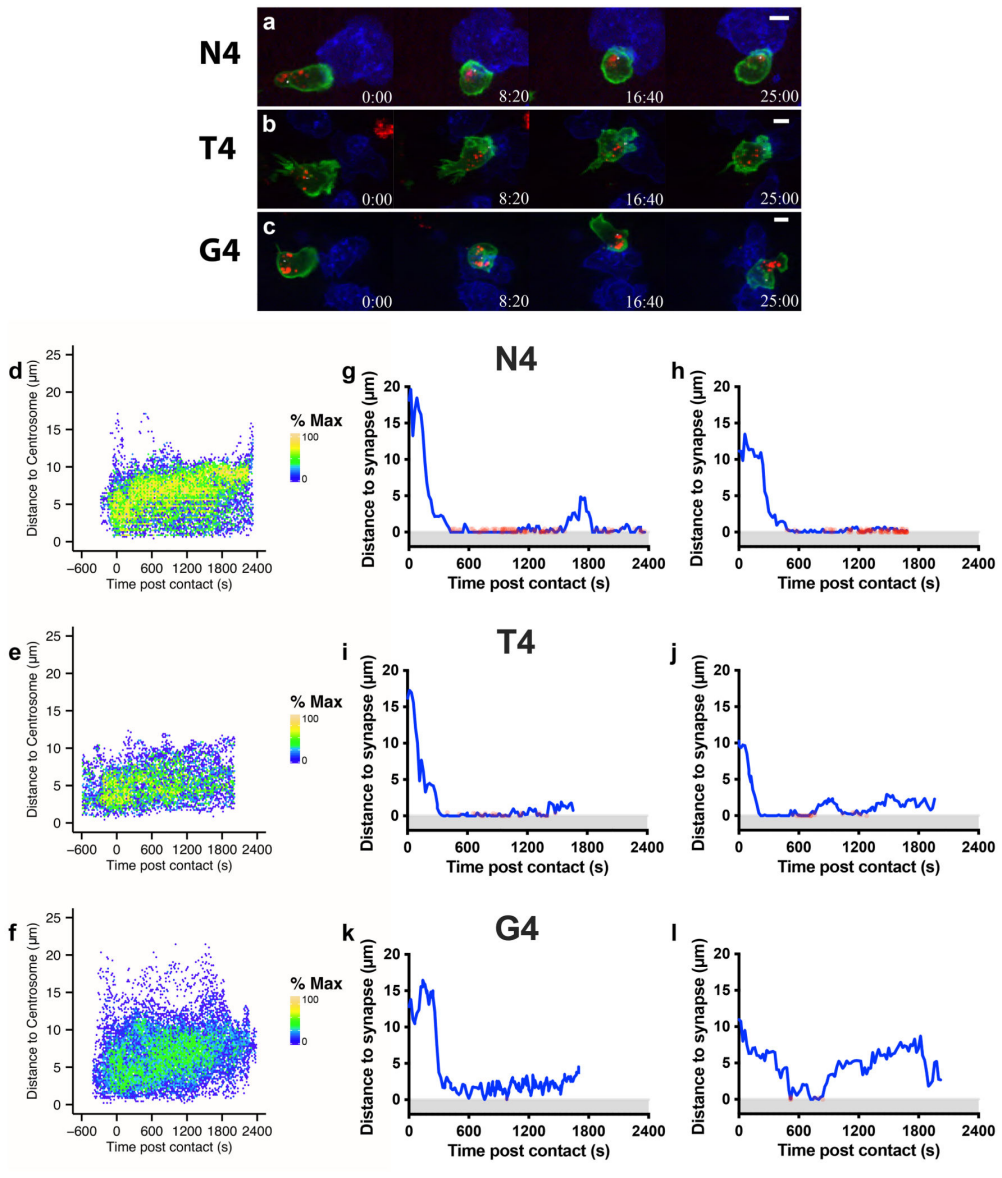
Prolonged docking of the centrosome to the synapse promotes granule delivery to the synapse. GzmB-TdTomato (red) OTI CTL expressing Lifeact-EGFP (green) and BFP- PACT (white sphere) interacting with EL4 (blue), pulsed with N4, T4, or G4 peptides. (a-b) Representative max-intensity projection timeseries of OTI CTLs encountering APL pulsed targets. Scale bars = 5μm, time min:sec post contact with target. (d-f) Normalised heatmap of granule to centrosome distances from segmented CTL target interactions shown in Figure 3b. Colour scale represents the density of granules relative to the maximum density on each plot. Total granule to centrosome distances measured (d) n=15192, (e) n=20343, (f) n=16443. (g-l) R was used to filter granules for concomitant centrosome docking and granule delivery (<0.5μm of the synapse) and plot them as red spots on a blue trace showing centrosome distance from the synapse. 2 representative cells are shown per APL, from a total of N4=10, T4=9, G4=10.

Previous work investigating the impact of APL on centrosome and granule polarisation in fixed conjugates suggested that lower signal strength led to centrosome but not granule polarisation to the synapse (Jenkins et al., 2009). We therefore followed the polarisation of the centrosome and granules using 4D imaging (Figure 4, Video 3). To determine if clustering of granules around the centrosome was impaired with weak TCR signal strength, we measured the distance from each granule to the centrosome as they polarised toward the synapse (Figure 4a-c). We found that with strong signals, granules behaved similarly to each other, keeping specific distances from the centrosome over time (high density of granules exhibiting uniform behaviour indicated by yellow in Figure 4d). In contrast, with reduced signal strength, we found a more heterogeneous distribution of granule to centrosome distance (lower density of granules exhibiting any particular behaviour indicated by green/blue in Figure 4e-f). To track both granules and the centrosome we classified both as docked when <1 μm from the synapse (Figure 4g-l). Using the strongest signal strength (N4) 90% polarised both centrosome and granules together, while with the weakest G4 80% did so. Thus, granule delivery via centrosome docking could be achieved by CTLs regardless of stimulation strength. Overall 93% of CTLs in which centrosome docking was seen, also showed concomitant granule delivery of at least 1 granule to within <0.5μm of the synapse, demonstrating that this pattern is shared across stimuli (Supplementary Figure 3). Hence the increased frequency of centrosome docking with high affinity ligand (N4) also increased a co-ordinated delivery of granules to the synapse.

### Ca^2+^ flux precedes uropod retraction and centrosome docking

**Figure 5:**
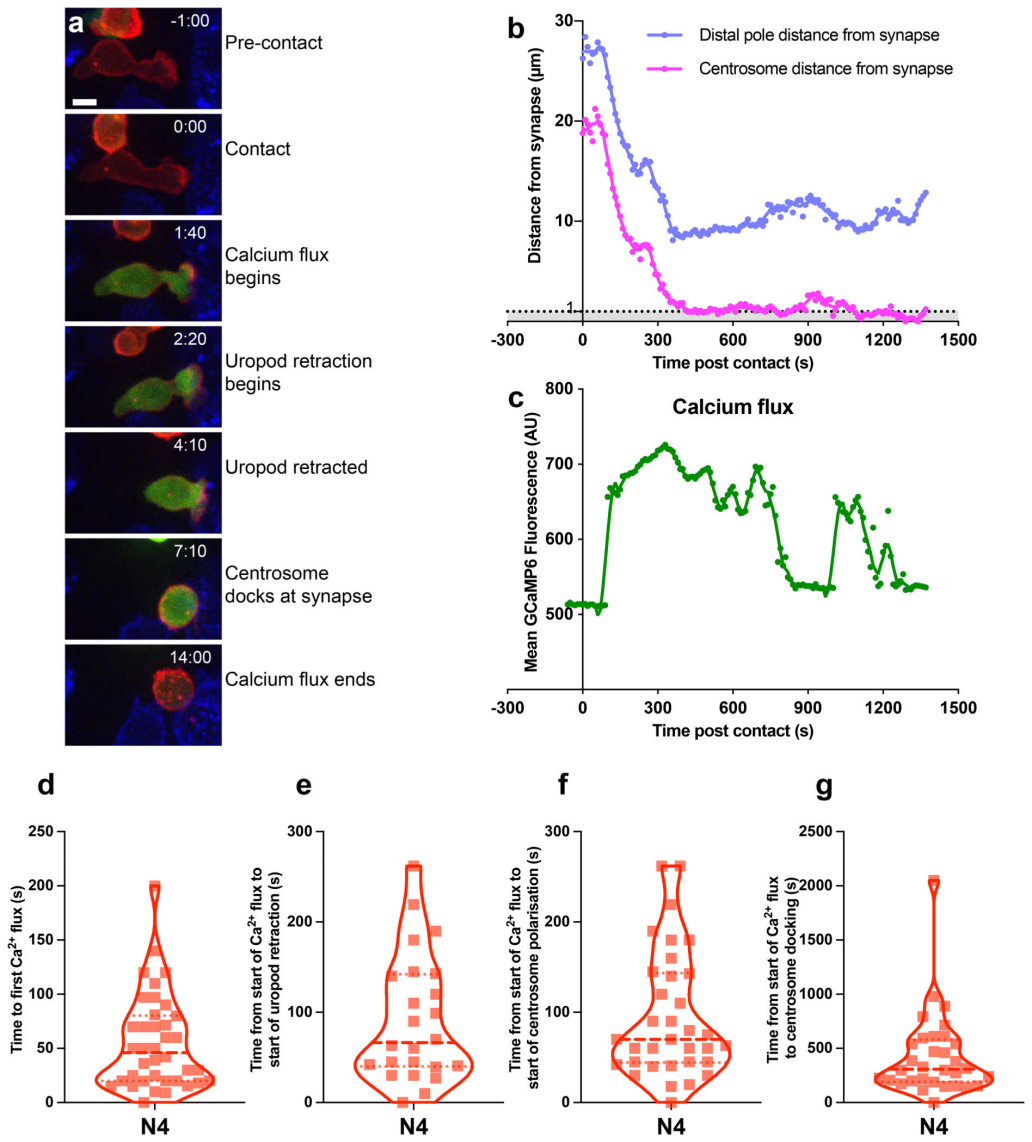
Calcium flux precedes centrosome polarisation and uropod retraction OTI CTL expressing GCAMP6m (green), Lifeact-mApple (red) and RFP-PACT (red sphere) interacting with EL4 (blue), pulsed with N4. (a-b) Representative max-intensity projection timeseries of OTI CTL encountering N4 pulsed target. Scale bars = 5μm, time min:sec post contact with target. (b-c) Example cell from (a) segmented with Imaris to measure: (b) the distance of the distal pole (blue) and centrosome (red) to the synapse. (c) The mean GCaMP6m fluorescence within the CTL. (d-g) Violin plots of (d) time from contact to first calcium flux, n=38, the time from the start of the calcium flux to; (e) start of uropod retraction (n=24), (f) start of centrosome polarisation toward synapse (n=34), (g) centrosome docking at the synapse (n=32).

As Ca^2+^ flux is required for both centrosome polarisation (Yi et al., 2013) and granule release (Maul-Pavicic et al., 2011) we asked how calcium flux relates to centrosome polarisation. OTI CTL were transfected with the GCaMP6 Ca^2+^ biosensor (Chen et al., 2013) before imaging with N4-presenting fluorescent- EL4 (Figure 5a-c, Video 4). We found that 42% of CTL generated a Ca^2+^ flux within 30s, and all CTL generated an initial Ca^2+^ flux with a mean time of 55s (median 46s) (Figure 5d). In those CTL where a uropod could be identified, we noted that it retracted soon after the first Ca^2+^ flux (mean 91.7s, median 66.5s), closely followed by the start of centrosome polarisation (mean 94s, median 70s) (Figure 5e,f). In 50% of CTL, the centrosome docked within 5 minutes of the initiation of the first Ca^2+^ flux, with a mean time of 428s (Figure 5g). This matched well with previous observations calculated from the time of first contact. Hence Ca^2+^ flux precedes uropod retraction and centrosome docking.

**Figure 6:**
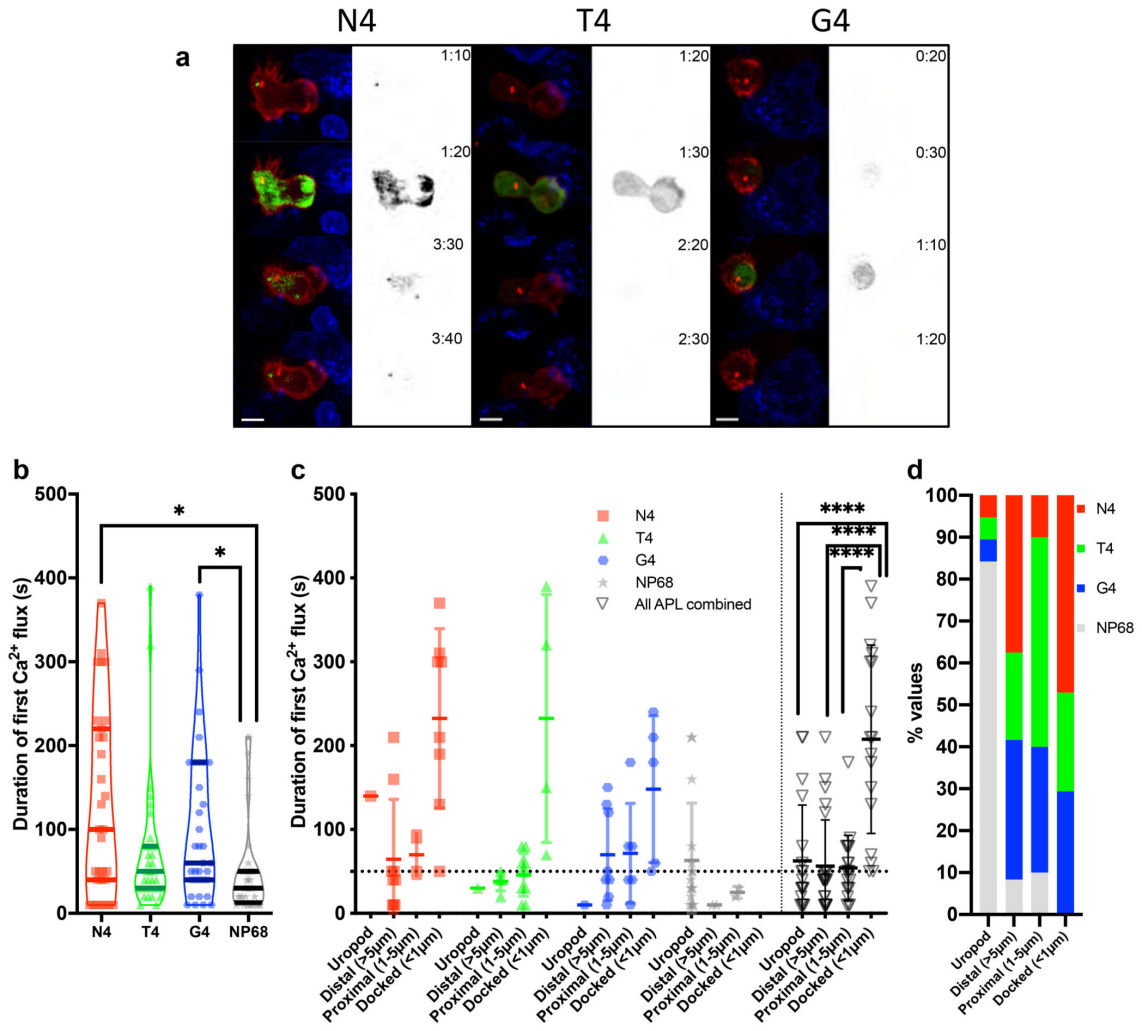
Duration of initial calcium fluxes are increased with higher affinity ligands OTI CTL expressing GCAMP6m (green), Lifeact-mApple (red) and RFP-PACT (red sphere) interacting with EL4 (blue), pulsed with N4, T4 or G4. (a) Representative max-intensity projection timeseries of OTI CTL encountering APL pulsed targets. Scale bars = 5μm, time min:sec post contact with target (Right, monochrome) fluorescence in the GCaMP6 channel. (b) The duration of the first increase in GCAMP6 fluorescence within an interaction (N4 n=31, T4/G4 n=35, NP68 n=36). Bars show medians with quartiles (c) duration of the first increase in GCaMP6 fluorescence within an interaction (n=20/APL downsampled from b). Measurements have been grouped in (c, d) by the APL presented by the target (symbol/colour) and by the closest approach of the centrosome to the target membrane. Bars show mean ±SD. (d) All data from (c) combined to show the percentage of cells for each APL with a given centrosome position. Statistics: Bonferoni corrected Mann-Whitney test *p<0.05, **p<0.01, ****p<0.00001.

We next asked how the strength of signal varied these responses (Figure 6a, Video 5). Quantitation of 4D imaging showed that as TCR signal strength increased, the median duration of the primary calcium flux also increased, from 30s for the null peptide NP68 to 60s for weak (G4) and 100s for strong (N4) TCR signalling (Figure 6b). Hence increasing TCR signal strength increased the frequency of a prolonged primary calcium flux.

We examined the relationship between the duration of the first Ca^2+^ flux and centrosome docking by identifying the closest centrosome-to-synapse position within a 40-minute window (Figure 6c). CTL centrosomes were classed as in the uropod; distal (>5μm); proximal (1-5μm) or docked (<1μm) (Figure 6c,d). CTL in which the centrosome was only ever observed in the uropod or >1 μm from the synapse showed initial calcium fluxes with means of ∼60s. In CTL where centrosome docking was successful the mean Ca^2+^ duration was 207s (median 190s) and always >50s (Figure 6c). Although CTLs from each stimulation condition could generate Ca^2+^ fluxes of >50s, a higher proportion of CTLs stimulated with N4 achieved centrosome docking (Figure 6d). These data suggest that increased signal strength increases the duration of Ca^2+^ flux and the frequency of centrosome docking. Thus, increased strength of signal leads to an increased frequency of CTL with longer dwell times, Ca^2+^ fluxes and resultant centrosome and granule polarisation, all favouring CTL mediated killing.

## Discussion

### Increasing TCR signal strength increases the killing efficiency of a target population

Many different studies have found that increasing the strength of TCR pMHC affinity increases killing of target cells as measured by target cell death or CTL degranulation. However how the changing strength of TCR signalling controls the cell biology of CTL killing has remained largely unexplored.

Most previous investigations have used end point analyses of conjugation efficacy, including microscopy and flow cytometry of fixed CTL target conjugates (Jenkins et al., 2009; Palmer et al., 2016; Yachi et al., 2006). Although imaging suggested an increased conjugation frequency with higher TCR affinity, the trend was weak and could be explained by an inability to distinguish long-lived TCR-dependent interactions from short-lived target sampling as observed with CTL cultured with NP68 presenting targets. A much stronger impact of TCR signal strength on conjugation frequency was seen using flow cytometry (Palmer et al., 2016). However, as the authors pointed out, the strength of adhesion may affect the estimation of the frequency of conjugated cells when using flow cytometry arguing for a role of TCR signal strength in the formation of a stable synapse. Combining Flow cytometry with a time course, showed the percentage of conjugated cells rapidly increased over the first 15 minutes with high affinity (N4) compared to low affinity (G4) ligands; however, conjugates frequencies equalised by 30 minutes.

Using live cell imaging, we now find that lower strength interactions (that give rise to asynchronous activation) decreased the dwell times of CTL with targets. Hence, as signal strength increases the probability of a CTL-target interaction being long-lived increases. Consequently, high affinity interactions result in rapid and synchronous conjugate formation, while lower affinity interactions require longer to form the same number of conjugates in a bulk population.

Interestingly, dwell time of naïve CD8 T cells has also been shown to increase with higher affinity TCR ligands (Le Borgne et al., 2016; Moreau et al., 2012; Ozga et al., 2016; Zahm et al., 2017). It is argued this prolonged dwell time allows naïve T cells to maximise the activation signals received from the antigen presenting cell and mount the stronger peripheral responses seen with stronger TCR signals. Our current study does not address the possibility that multiple weak affinity interactions can accumulate in CTL leading to higher levels of intracellular activation. Likewise, *in vivo* studies with 2-photon microscopy of CTL killing virally infected cells were also unable to determine whether single CTL killing rates increased or decreased after sequential target encounters (Halle et al., 2016).

### Strong TCR signalling promotes the loss of charge and resulting depletion of actin from the synapse

Many studies have shown dynamic changes in the actin cytoskeleton across the immune synapse (Blumenthal and Burkhardt, 2020). Early studies suggested weak signals reduced actin polymerisation at the synapse, (Palmer et al., 2016) although actin depletion could be observed in CTL with both N4 and G4 generated conjugates (Jenkins et al., 2009). More recent data has revealed a clear molecular link between TCR signalling and actin depletion, whereby TCR ligation triggered changes in the phosphoinositide composition with a loss of PIP2 and decreased membrane-charge leading to depletion of actin across the synapse (Gawden-Bone et al., 2018). This predicts that stronger signals should generate greater actin depletion. Testing this hypothesis, we found that both loss of membrane charge and actin depletion were greater with higher affinity ligand (N4). Consistent with our findings, a recent study in CD4+ T cells also found that stronger TCR signals resulted in decreased levels of PIP2 compared to weaker TCR signals (Hawse and Cattley, 2019).

### Increasing TCR signal strength promotes centrosome polarisation to the synapse

Centrosome polarisation and docking within the synapse is a crucial step in the killing process (Stinchcombe et al., 2006; Stinchcombe et al., 2015). The accumulation of DAG at the synapse has been shown to trigger centrosome polarisation and be required for CTL killing, with centriole deletion (formed after Sas4/p53 deletion) resulting in reduced killing (Quann et al., 2009; Tamzalit et al., 2020). Dynein at the immune synapse is thought to play a role in generating the forces required for centrosome translocation (Combs et al., 2006; Stinchcombe et al., 2006; Yi et al., 2013). The movement of the centrosome has been shown to be biphasic with dynein-mediated end-on capture shrinkage of “pioneer” microtubules drawing the centrosome into the proximity of the synapse rapidly, before it slowly moves to associate with the membrane (Yi et al., 2013). Other proteins involved in microtubule dynamics have also been implicated, including the kinesin-4 protein KIF21B that facilitates centrosome polarisation by limiting the growth of microtubules (Hooikaas et al., 2020). Although studies using N4 stimulation have described similar rates of centrosome polarisation (Ritter et al., 2015; Yi et al., 2013), whether the rate of centrosome polarisation changes according to TCR signal strength had not been explored.

In this study we determined the speed with which the centrosome moved towards the synapse by measuring the centrosome to synapse distance over time. We found that as TCR signal strength increased the speed of centrosome movement towards the synapse did not change, although the rate at which a CTL population achieved centrosome docking at the synapse increased. This indicates that the underlying mechanics of centrosome polarisation are independent of TCR signal strength. However, the rate at which centrosome polarisation was triggered within the CTL population increased with signal strength. We noted that the centrosome polarised and retracted from the synapse multiple times with weaker TCR signals, contributing to a much greater heterogeneity of centrosome docking within the population. Thus, the success of centrosome docking paralleled the success of killing. Our data therefore support a critical role for centrosome docking in killing.

### Docking of the centrosome promotes simultaneous delivery of granules to the synapse

Our results suggest that granules cluster around the centrosome for the first 5 minutes of an interaction (Figure 4), but then the majority disperse, leaving a small percentage close to the centrosome. As TCR signal strength increased, granules maintained a more uniform distance from the centrosome, as indicated by the yellow intensity (Figure 4d-f). Previous studies using imaging of fixed cells suggested that granule clustering is impaired with weak TCR signals (Beal et al., 2009; Jenkins et al., 2009); while live imaging showed mean granule distance from the centrosome gradually increased ~2-3 minutes after centrosome docking (Ritter et al., 2015). Our data now resolves these results by showing that increasing TCR signal strength increases the proportion of cells in which coordinated delivery of granules to the synapse is successful.

### Ca^2+^ flux precedes uropod retraction and centrosome docking

Early work showed Ca^2+^ flux is important in CTL recognition and killing of target cells (Lancki et al., 1987; Takayama and Sitkovsky, 1987), with Ca^2+^ flux preceding cell rounding (Donnadieu et al., 1994; Negulescu et al., 1996), and delivery of the lethal hit (Poenie et al., 1987; Zhou et al., 2018). However, the timing relative to centrosome polarisation and docking had been overlooked. We now find that the initial CTL Ca^2+^ flux in response to strong TCR signal (N4) occurs rapidly upon target contact, before uropod retraction and centrosome docking at the synapse. We also noted that as TCR signal strength increased, so too did the predominance of CTL displaying a prolonged Ca^2+^ flux rather than many short oscillating fluxes, agreeing with single cell measurements of Ca^2+^ flux distinguishing these populations (Chen et al., 2010; Christo et al., 2015; Dong et al., 2017; Frick et al., 2017; Le Borgne et al., 2016; Liu et al., 2014; Wulfing et al., 1997; Xia et al., 2018).

The role of Ca^2+^ in centrosome polarisation has been controversial (Ritter et al., 2013), and previous studies have been limited to using Ca^2+^ chelation or fluorophores best suited to bulk populations. The availability of GCaMP6 and live imaging provided a new opportunity to investigate the links between Ca^2+^ fluxes and centrosome polarisation. We found multiple Ca^2+^ fluxes during a single interaction, noting a Ca^2+^ flux of at least 50s was necessary, if not sufficient, for centrosome docking regardless of signal strength. Furthermore, we found that increasing TCR signal strength increased the proportion of interactions leading to a Ca^2+^ flux >50s. It is possible that phosphorylation of LAT Y132, which plays a role in ligand discrimination by driving more rapid Ca^2+^ fluxes, contributes to this fine tuning (Lo et al., 2019). Thus, our findings suggest that stronger signals reduce the heterogeneity of the Ca^2+^ fluxes within the population favouring centrosome docking.

### A rate-based mechanism for T cell activation controls polarisation

Recent single-cell studies on the activation of naïve CD8+ T cells have revealed that the changes within populations are controlled by the rate at which conserved TCR signalling pathways activate within individual cells (Ma et al., 2020). Likewise, the transcriptional trajectory following TCR activation is conserved regardless of the strength of TCR signal, but the rate at which individual cells initiate activation increases with increasing strength of signal (Richard et al., 2018). Consequently, within a population, increasing signal strength will increase the number of cells that have activated. These results suggest that both at the signalling and transcriptional levels, naïve T cell activation pathways are conserved, but the rate at which individual cells initiate their progress along these pathways changes with strength of signal. Our results examining activated CD8+ T cells (ie CTL) now show that TCR signal strength controls efficient CTL killing of a target population through modulating heterogeneity within the population at multiple distinct stages. These include dwell time, initial Ca^2+^ flux duration, membrane specialisation, centrosome docking, and granule clustering and delivery to the synapse. The proportion of CTL engaged in each step of this process was greater with increased TCR signal strength. Consequently, strong signals gave rapid and homogeneous responses while weak signals generated much more heterogeneity, reducing the rate of target killing. We found that those cells that reached full activation showed centrosome docking and granule polarisation regardless of the initial signal strength. Furthermore, pathways leading to successful granule delivery, such as the speed of centrosome movement, were conserved across all signal strengths. Our results are consistent with an emerging model of signal strength controlling the rate of progression along conserved pathways, as seen for transcriptional activation and intracellular signalling in naïve T cells (Ma et al., 2020; Richard et al., 2018). This model explains the delayed kinetics of weak ligand responses in bulk populations that were often interpreted as slowed rather than asynchronous responses.

## Materials and Methods

### DNA constructs

BFP-PACT in the pTagBFP-C (Evrogen) vector, RFP-PACT (Gillingham and Munro, 2000), mApple-LifeAct-7 (Addgene plasmid #54747), EGFP-LifeAct-7 (Addgene plasmid #54610) (Riedl et al., 2008) were as used in (Ritter et al., 2015). LAMP-1-mApple, EGFP-Kras8+ (Yeung et al., 2008) were gifts from M. Davidson and Sergio Grinstein respectively. GCaMP6m was a gift from Douglas Kim (Addgene plasmid #40754) (Chen et al., 2013).

### Mice

C57BL/6 (B6)-OTI Rag2^-/-^ (B6.129S6-Rag2tm1Fwa Tg(TcraTcrb)1100Mjb) mice referred to as OTI mice, and GzmB-TdTomato OTI mice, referred to as GzmB-TdTom OTI, gifted from Claude Boyer (Mouchacca et al., 2013) were bred and maintained in specific pathogen free conditions. Experiments were carried out under Project Licence PPL 70/8590. This research has been regulated under the Animals (Scientific Procedures) Act 1986 Amendment Regulations 2012 following ethical review by the University of Cambridge Animal Welfare and Ethical Review Body (AWERB).

### Cell culture

Naïve OTI splenocytes were stimulated with 10 nM SIINFEKL (Cambridge Bioscience) for 3 days in RPMI 1640 medium (Sigma-Aldrich, Cat# 1640) with 10% FBS (LabTech, Cat# FBS-SA), 50 mM β-Mercaptoethanol (Thermo Fisher Scientific, Cat# 31350010), 10 U/ml recombinant murine IL-2 (Peprotech, Cat# 212-12), 2 mM L-Glutamine (Sigma-Aldrich, Cat# G7513-100ML), 1mM sodium pyruvate (Thermo Fisher Scientific, Cat# 11360070) and 50 U/ml penicillin and streptomycin (Sigma-Aldrich, Cat# P0781-100ML). Cells were washed and seeded into fresh media on a daily basis from 3 days post stimulation. Target EL4 and fluorescent EL4 cells (Ritter et al., 2015) were maintained in DMEM (Sigma-Aldrich, Cat# D5030-10X1L) supplemented with 10% FBS and 2mM L- glutamine.

### Antibodies and reagents

OVA_257-261_ SIINFEKL along with all APL (Q4; SIIQFEKL, Q4R7, SIIKFERL, T4, SIITFEKL, Q4H7, SIIQFEHL, G4, SIIGFEKL) and the NP68 ASNENMDAM were obtained from Cambridge Bioscience. Recombinant mouse ICAM- 1/CD45 Fc Chimera Protein Recombinant ICAM1 was obtained from R&D systems (Cat# 796-IC). For degranulation assays, Rat PE-anti-mouse CD107a (1D4B) (Cat# 12-1071-83) and Rat APC-anti-mouse CD8α (53-6.7) (Cat# 100712) were from Biolegend. Phospho-tyrosine was bound with Mouse Anti-Phosphotyrosine Platinum 4G10 from Merck/Millipore (Cat# 05-1050) and detected by Alexa Fluor 647 donkey anti-mouse (H+L) from Thermo Fisher (Cat# A32787). Alexa Fluor 555-Phalloidin was from Invitrogen (Cat# A34055).

### Killing assay

Cytotoxic function was assessed with the Promega Cytotox 96 Non-radioactive cytotoxicity assay (Promega, Cat# G1780). EL4 cells were pulsed for 1 h with 1 μM APL at 37 °C 8% CO2, washed three times in phenol-red-free RPMI, 2% BSA (Sigma-Aldrich, Cat# A7906-500G) (killing assay medium) and 10^4^ pulsed EL4 cells were resuspended with CTL at effector to target ratios shown. After 2 h 37°C 8% CO2, supernatant was collected and LDH activity at room temperature (RT) was detected by absorbance reading after 30 min exposure at 490 nm with a VERSAmax spectrophotometer (Molecular devices). %Lysis was calculated as: ((effector induced cell death – blank)-(effector spontaneous death – blank) – (target spontaneous death))/ ((Lysed targets – lysis control).

### Degranulation assay

EL4 cells were pulsed for 1 h with 1 μM APL at 37 °C 10% CO2 before washing into RPMI,10% FBS. CTL and EL4 were mixed 1:1 and plated in triplicate at 2x10^5^/ml in 200μl per well with 4 μg/ml PE-anti-LAMP-1 (Biolegend clone 1D4B). At the stated timepoints cells were washed with ice cold PBS before fixing with 2% PFA (Electron Microscopy Sciences, Cat# 15710-S), PBS for 10 min RT. Cells were then washed in PBS, 1% FBS (FACS buffer) and left at 4 °C until all timepoints had been collected. Cells were stained with APC-anti- mouse CD8α (Biolegend clone 53-6.7) for 30 min 4 °C before data acquisition with a BD FACSCalibur. CTL were gated on FSCvSSC for lymphocytes, then CD8^+^ cells before analysing the % LAMP-1^+^ and geometric mean PE fluorescence.

### Immunofluorescence imaging of actin in fixed cells

24 h before the experiment and 5-8 days post stimulation, 5x10^6^ OTI CTL were transfected with 5μg of DNA containing the EGFP-Kras8+ probe using the Mouse CD8 T cell nucleofection kit (Amaxa). EL4 expressing Farnesyl-5- TagBFP2 were pulsed with 1μM APL for 1 hour 37°C, washed three times and resuspended at 10^6^ cells/ml together with 10^6^ cells/ml OTI cells expressing EGFP-Kras8+ (1:1 ratio). CTLs and targets were allowed to form conjugates at 37°C for 5 minutes, before being placed on 5-well slides (Hendley, Cat# P299) using a cut-off pipette tip and incubated at 37°C, 10% CO2 for the times shown. Conjugates were fixed in 4% PFA at RT for 5 minutes, washed in PBS, quenched in PBS, 50mM ammonium chloride (Sigma-Aldrich, Cat# 254134), for 10 minutes, permeabilised with 0.2% Triton X100 (Sigma-Aldritch, Cat# T8787-100ML), PBS for 5 min RT, and blocked with PBS,1% BSA for 1h 4°C before labelling with Mouse Anti-Phosphotyrosine Platinum 4G10 (Merck/Millipore Cat# 05-1050) and donkey α -mouse (H+L) 647 Alexa Fluor secondary antibody (Thermo Fisher Scientific, Cat# A32787) with Phalloidin- Alexa-555 (Invitrogen, Cat# A34055). Samples were mounted in ProLong Diamond Antifade Mountant (Thermo Fisher Scientific Cat# P36961) with a No.1.5 coverslip and imaged at RT using an Andor system, with an Olympus IX81S1F-3-5 body, Piezo Z and motor XY stage control (H117E2IX), Yokogawa CSU-X1 spinning disk and iXon Ultra 888 camera (Andor) with Andor IQ3 software (Olympus Plan Appochromat 100x 1.4n.a. oil objective and z stepdistance of 0.2μm). Images were analysed using Bitplane Imaris 8.3.1 and *en face* images generated using an oblique slice of 3μm thick which was exported to ImageJ for further quantification. Intensity profiles were acquired using the line tool analysis and %synapse area depleted was calculated as 100x(1-(area of synapse above background threshold)/(area of synapse above threshold with gaps filled in).

### Live Microscopy

24 h before imaging and 5-8 days post stimulation, 5x10^6^ CTL were transfected with 5-16 μg DNA using the Mouse CD8 T cell nucleofection kit (Amaxa, Cat# VPA-1006). EL4 expressing either Farnesyl-5-TagBFP2 or Mem-TagiRFP670 were used as targets and pulsed for 30 min with 1 μM peptide at 37 °C 8% CO2 before washing into serum-free DMEM and applying to 1 μg/ml murine-ICAM- 1 coated 35 mm glass bottomed culture dishes at 6.5x10^5^/ml. After 5 min to adhere, unbound targets were washed clear with phenol-red free T cell medium plus 25 mM HEPES and the dish loaded onto the microscope. Approximately 2x10^6^ nucleofected CTL were dropped onto the dish and imaging began within 5 min.

Imaging of CTL:Target interactions used the system described above with an Olympus Universal Plan Super Apochromat 60x 1.3NA silicone oil objective, and an OKOLAB stage incubator to maintain a 37 °C temperature and ∼5% CO_2_ atmosphere. Each z-plane was separated by 0.8 μm with the z-dimension ranging from 16-20 μm and image stacks taken every 5-20 s for 20-40 min. Fluorophores were excited with 405, 488, 561 and 640 nm lasers in each plane. Data was captured with the iQ3 software (Andor) before visualising and analysing with Imaris (Bitplane).

### Live cell object based image analysis

To measure Ca^2+^ flux and follow centrosome to granule and or synapse distances over time, these structures were segmented with the Bitplane Imaris software. In brief, the boundaries of the CTL and target were segmented using the Imaris surface function on the LifeAct signal for the CTL and the relevant signal for the target membrane as the target cell. Cytotoxic granules were modelled using the spots function on the RFP-fluorescent signal and the centrosome was modelled with the spots function on the PACT signal. The Imaris cell function was then used on these boundaries to measure the intracellular distance from the immune synapse to the centrosome or the centrosome to the cytotoxic granules. Where just the centrosome distance to the synapse was measured, the synapse was represented as a surface where the target and CTL models overlapped and the shortest distance calculated. In contrast where centrosome to granule distances were concurrently measured it was required to convert the synapse surface to spots and manually limit these to provide an equivalent shortest distance, but with repeat measurements to account for the introduced human error. Rstudio 1.0.136 (Rstudio, Inc) with R version 3.0.2 (R Foundation for Statistical Computing) and ggplot2 (Wickham and SpringerLink, 2009) was used to plot granule to centrosome distances as a hexbin density plot, as well as filter centrosome and granule to immune synapse distance as <0.5μm for Figure 4 and Supplementary Figure 3. Calcium fluorescence was calculated as the mean GFP intensity within the bounds of the CTL surface. For greater detail see (Frazer et al., 2017).

### Live cell manual analysis

The primary calcium flux length was measured from the first frame the GCaMP6m fluorescence visibly exceeded background until the frame where it returned to this intensity. Centrosome to synapse distances were measured within the central plane of the PACT fluorescence from the centre of this structure to the target fluorescence using the Imaris distance tool. Interactions were assumed when the CTL LifeAct signal appeared to touch the target membrane and dwell time measured until this ceased, this signal was also used to determine the presence and retraction of the uropod.

#### Statistics

A two-tailed Bonferroni corrected Mann-Whitney test was used for all statistical analyses.

## Supplementary Material

Supplementary Figures

## Summary

Frazer *et al* use 4D imaging to reveal that while the intracellular pathways orchestrating killing within CTL are conserved, the rate of initiation along these pathways varies with TCR signal strength.
