# Supplementary material for: Signal strength controls the rate of polarisation within CTLs during killing
Source: J Cell Biol. Author manuscript; Available in PMC 2021 Oct 4. (PMC8302442; doi:10.1083/jcb.202104093)
Supplement: Supplementary Figures [file EMS131835-supplement-Supplementary_Figures.pdf]

max-intensity projection timeseries of OTI CTL encountering N4 pulsed target. Scale bars = 5μm, time min:sec post contact with target. (b-c) Example cell from (a) segmented with Imaris to measure: (b) the distance of the distal pole (blue) and centrosome (red) to the synapse. (c) The mean GCaMP6m fluorescence within the CTL. (d-g) Violin plots of (d) time from contact to first calcium flux, n=38, the time from the start of the calcium flux to; (e) start of uropod retraction (n=24), (f) start of centrosome polarisation toward synapse (n=34), (g) centrosome docking at the synapse (n=32).

Figure 6 Duration of initial calcium fluxes are increased with higher affinity ligands

OTI CTL expressing GCAMP6m (green), Lifeact-mApple (red) and RFP-PACT (red sphere) interacting with EL4 (blue), pulsed with N4, T4 or G4. (a) Representative max-intensity projection timeseries of OTI CTL encountering APL pulsed targets. Scale bars = 5μm, time min:sec post contact with target (Right, monochrome) fluorescence in the GCaMP6 channel. (b) The duration of the first increase in GCAMP6 fluorescence within an interaction (N4 n=31, T4/G4 n=35, NP68 n=36). Bars show medians with quartiles (c) duration of the first increase in GCaMP6 fluorescence within an interaction (n=20/APL downsampled from b). Measurements have been grouped in (c, d) by the APL presented by the target (symbol/colour) and by the closest approach of the centrosome to the target membrane. Bars show mean ±SD. (d) All data from (c) combined to show the percentage of cells for each APL with a given centrosome position. Statistics: Bonferoni corrected Mann-Whitney test *p<0.05, **p<0.01, ****p<0.00001.

Supplementary Figures

Supplementary Figure 1. **Stronger TCR signal strengths increase CTL killing efficiency.**

(a) Killing assay. OTI CTL were incubated with APL, as shown, presenting EL4 at the shown CTL effector:Target (E) ratios and target lysis measured by LDH release, showing means ±SD of triplicate values for 1 representative

experiment of 6. (b) LAMP-1 degranulation assay, showing LAMP-1 signal from OTI CTL (gated on CD8) incubated with EL4 pulsed with APL (indicated by same colours as in (a)), or no EL4 (broken line) for 2.5h. (c) Geometric mean fluorescence and (d) percentage $LAMP1^+$ CTL against time showing means of triplicate samples ± SD from 1 representative experiment of 2.

Supplementary Figure 2, related to Figure 3b.

Centrosome speed is unaffected by TCR signal strength.

Segmented centrosome speed relative to the synapse measured across the duration of the interaction for n=10 (N4, G4) or n=9 (T4) independent CTL-target interactions, with each colour representing a different CTL.

Supplementary Figure 3, related to Figure 4 (g-l).

Centrosome and granule docking

GzmB-TdTomato OTI CTL expressing Lifeact-EGFP and BFP-PACT interacting with EL4, pulsed with N4, T4, or G4 peptides were segmented using Imaris to measure relative distances to the synapse. R was used to filter granules for concomitant centrosome docking and granule delivery (<0.5 μm of the synapse) and plot them as red spots (granules) on a blue trace (centrosome) showing centrosome distance from the synapse, from total CTL of N4=10, T4=9, G4=10. Representatives were taken from these for Figure 4g-l.

Video S1 relating to Figure 1a. Increasing TCR signal strength increases CTL dwell time.

OTI CTL expressing Lifeact-mApple (green) and RFP-PACT (green) interacting with EL4 (blue), pulsed with N4, G4 or NP68 peptides. APL relates to the max-intensity projection representative timeseries of OTI CTLs encountering targets shown in Figure 1. Scale bars = 5μm time min:sec from start of recording.

Video S2 relating to Figure 3a. **Increasing TCR signal strength increases centrosome docking at the synapse.**

GzmB-TdTomato (red) OTI CTL expressing Lifeact-EGFP (green) and BFP-PACT (white sphere) interacting with EL4 (blue), pulsed with N4, T4 or G4 peptides. APL relates to the representative timeseries max-intensity projection of OTI CTLs encountering targets shown in Figure 3a. Scale bars = 5μm time min:sec from start of recording.

Video S3 relating to Figure 4a-c **Prolonged docking of the centrosome to the synapse promotes granule delivery to the synapse.**
GzmB-TdTomato (red) OTI CTL expressing Lifeact-mApple (green) and BFP-PACT (red sphere) interacting with EL4-blue, pulsed with N4, T4 or G4 peptides. APL relates to the representative max-intensity projection timeseries of OTI CTLs encountering targets shown in Figure 4a-c. Scale bars = 5μm time min:sec from start of recording.

Video S4 relating to Figure 5a. **Calcium flux precedes centrosome polarisation and uropod retraction**
OTI CTL expressing GCAMP6m (green), Lifeact-mApple (red) and RFP-PACT (red sphere) interacting with EL4 (blue), pulsed with N4. Video relates to representative max-intensity projection timeseries of OTI CTL encountering N4 pulsed target in Figure 5a. Scale bars = 5μm, time min:sec post from start of recording.

Video S5 relating to Figure 6a **Duration of initial calcium fluxes are increased with higher affinity ligands**
OTI CTL expressing GCAMP6m (green), Lifeact-mApple (red) and RFP-PACT (red sphere) interacting with EL4 (blue), pulsed with N4, T4 or G4. Video relates to Figure 6(a) Representative max-intensity projection timeseries of OTI CTL encountering APL pulsed targets. Scale bars = 5μm, time min:sec from start of recording.

References

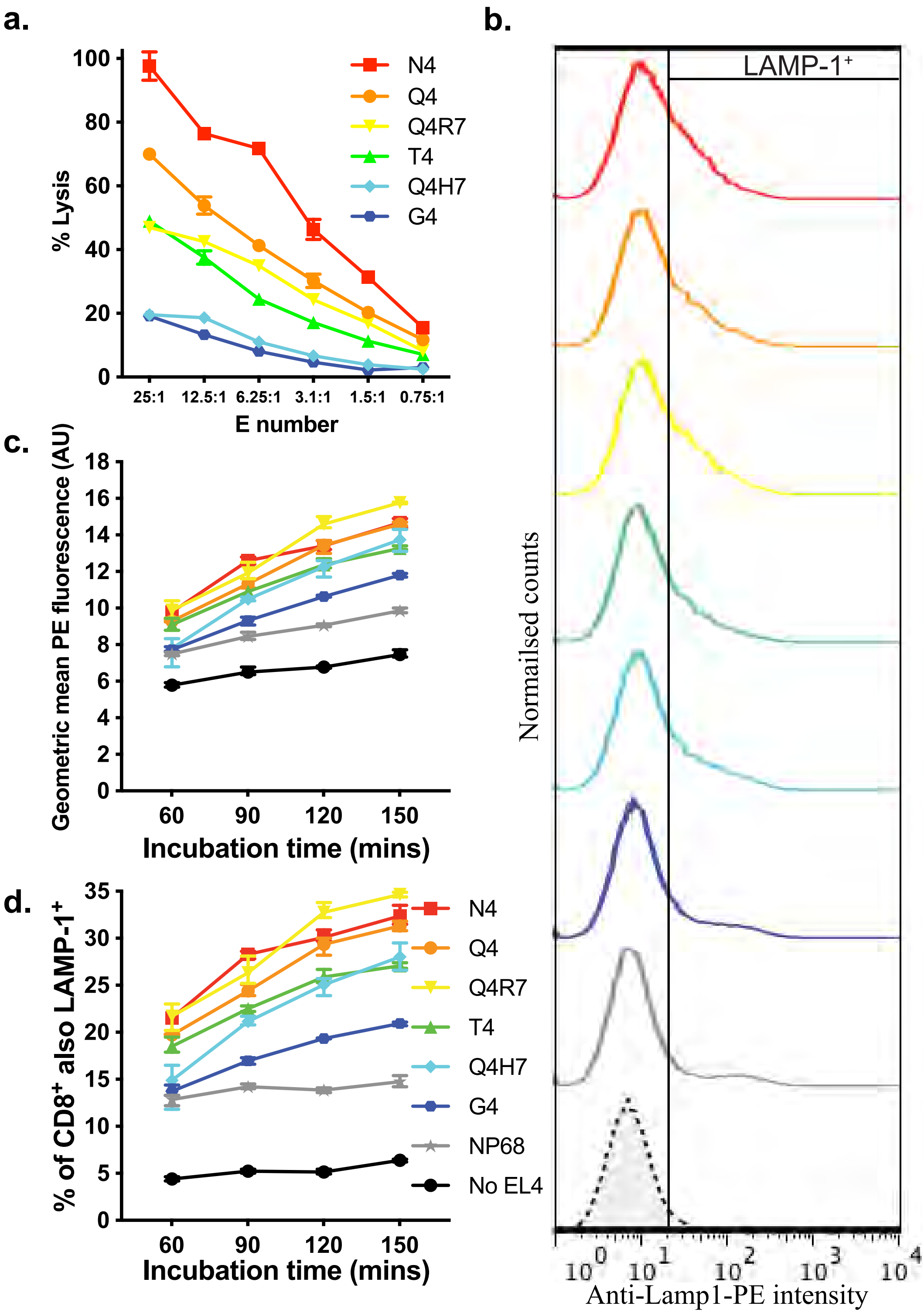

a.
b.
c.
d.
100
80
60
40
20
0
% Lysis
25:1
12.5:1
6.25:1
3.1:1
1.5:1
0.75:1
E number
N4
Q4
Q4R7
T4
Q4H7
G4
18
16
14
12
10
8
6
4
2
0
Geometric mean PE fluorescence (AU)
60
90
120
150
Incubation time (mins)
35
30
25
20
15
10
5
0
% of CD8+ also LAMP-1+
60
90
120
150
Incubation time (mins)
N4
Q4
Q4R7
T4
Q4H7
G4
NP68
No EL4
LAMP-1+
Normailsed counts
10^0
10^1
10^2
10^3
10^4
Anti-Lamp1-PE intensity

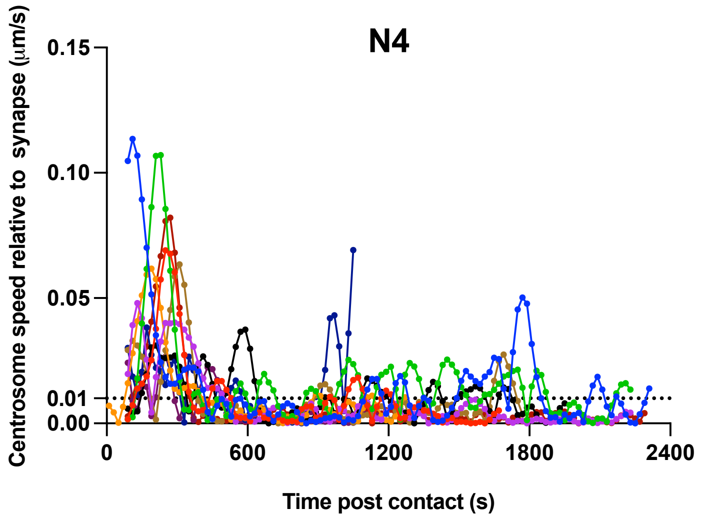
N4
Centrosome speed relative to synapse (μm/s)
0.15
0.10
0.05
0.01
0.00
0
600
1200
1800
2400
Time post contact (s)

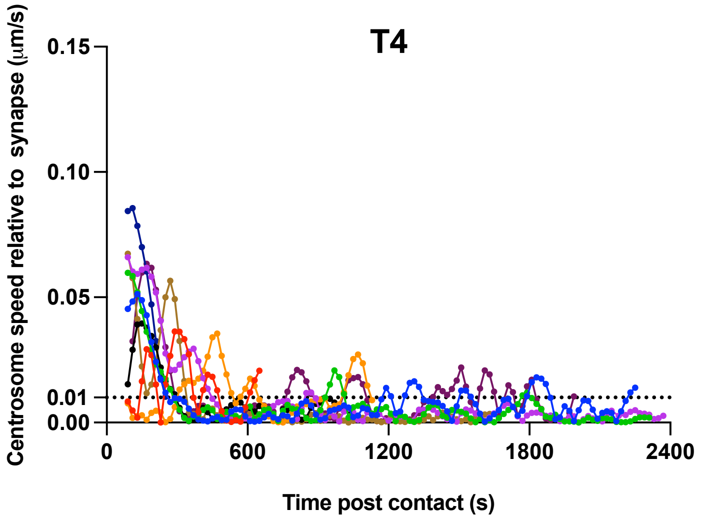
T4
Centrosome speed relative to synapse (μm/s)
0.15
0.10
0.05
0.01
0.00
0
600
1200
1800
2400
Time post contact (s)

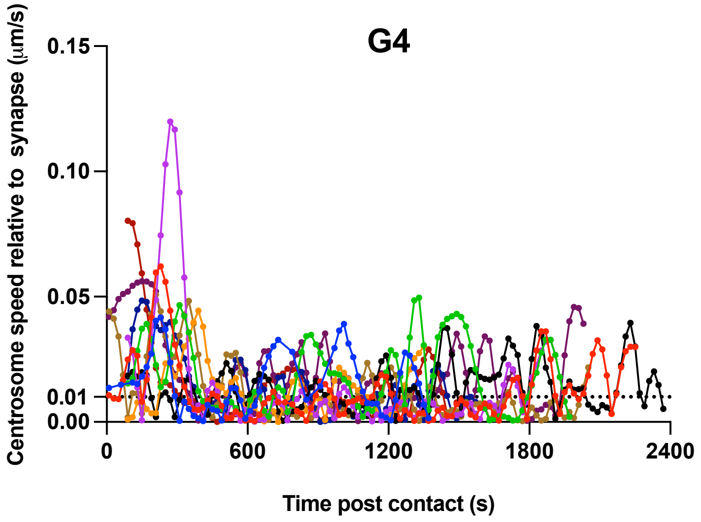
G4
Centrosome speed relative to synapse (μm/s)
0.15
0.10
0.05
0.01
0.00
0
600
1200
1800
2400
Time post contact (s)

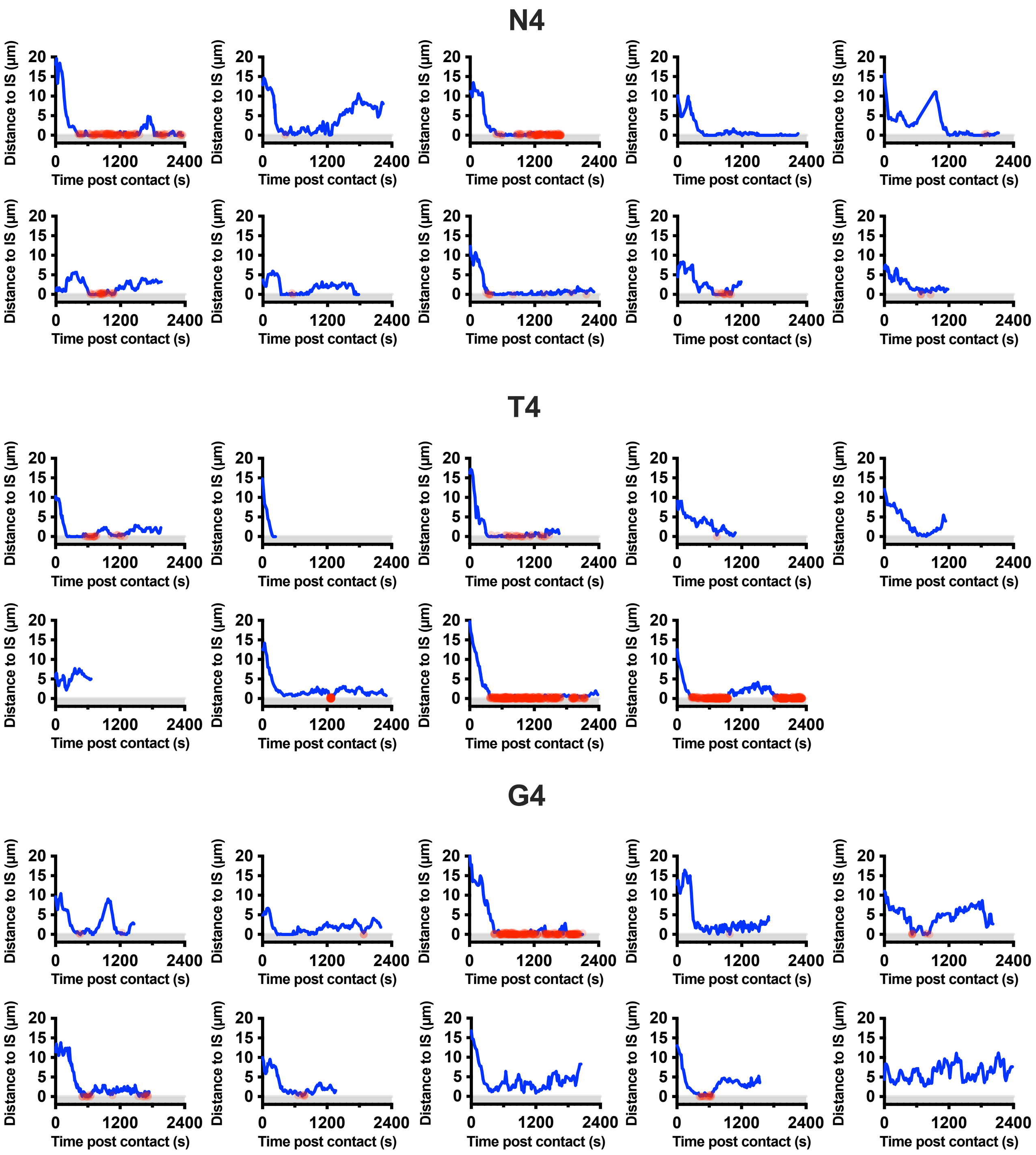
N4
T4
G4
Distance to IS (μm)
20
15
10
5
0
0
1200
2400
Time post contact (s)
